# Randomized Controlled Trial Evaluating Rotigotine Safety in PKD

**DOI:** 10.1016/j.ekir.2026.106682

**Published:** 2026-06-24

**Authors:** Audrey Dumont, Adrien Cour, Muriel Quillard-Murraine, Margaux Van Wynsberghe, Sophie Ruault, Sabrina Prod’Homme, Estelle Houivet, Gabriel Choukroun, Cédric Renard, Clémence Béchade, Amandine Claudinot, François Glowacki, Philippe Puech, Nell Marty, Virginie Buchbach, Nathalie Donnadieu, Elise Duhamel, Jean-Nicolas Dacher, Dominique Guerrot, Jérémy Bellien

**Affiliations:** 1 Department of Pharmacology, Université Rouen Normandie, Institut national de la santé et de la recherche médicale (INSERM) U1096, EnVI, Centre Hospitalier Universitaire (CHU) Rouen, Rouen, France; 2 Department of Radiology, Université Rouen Normandie, Centre Hospitalier Universitaire (CHU) Rouen, France; 3 Department of Biochemistry, Université Rouen Normandie, Centre Hospitalier Universitaire (CHU) Rouen, Rouen, France; 4 Innovation and Clinical Research Direction, Centre Hospitalier Universitaire (CHU) Rouen, Rouen, France; 5 Department of Biostatistics, Centre Hospitalier Universitaire (CHU) Rouen, Rouen, France; 6 Department of Nephrology, Dialysis and Transplantation, University of Picardie Jules Verne, MP3CV Laboratory, Centre Hospitalier Universitaire (CHU) Amiens, Amiens, France; 7 Department of Radiology, University of Picardie Jules Verne, Centre Hospitalier Universitaire (CHU) Amiens, Amiens, France; 8 Department of Nephrology, Normandie Université, UNICAEN, ANTICIPE U1086 INSERM-UCN, Centre Hospitalier Universitaire (CHU) Caen Normandie, Caen, France; 9 Department of Radiology, Normandie Université, UNICAEN, Centre Hospitalier Universitaire (CHU) Caen Normandie, Caen, France; 10 Department of Nephrology, Dialysis, Kidney Transplantation, and Apheresis, Centre Hospitalier Universitaire (CHU) Lille, Centre National de la Recherche Scientifique (CNRS) Institut national de la santé et de la recherche médicale (INSERM), UMR 1167 RID-AGE, Université Lille, Lille, France; 11 Department of Radiology, Université Lille, Centre Hospitalier Universitaire (CHU) Lille, Lille, France; 12 Department of Pharmacy, Centre Hospitalier Universitaire (CHU) Rouen, Université Rouen Normandie, Rouen, France; 13 Department of Radiology, Centre Hospitalier Universitaire (CHU) Rouen, Centre d'Investigation Clinique - Centre de Ressources Biologiques (CIC-CRB 1404), Institut national de la santé et de la recherche médicale (INSERM) U1096, EnVI, Université Rouen Normandie, Rouen, France; 14 Department of Nephrology, Centre Hospitalier Universitaire (CHU) Rouen, Institut national de la santé et de la recherche médicale (INSERM) U1096, EnVI, Université Rouen Normandie, Rouen, France

**Keywords:** ADPKD, rotigotine, safety

## Introduction

Autosomal dominant polycystic kidney disease (ADPKD) is the most common inherited kidney disorder and is mainly caused by pathogenic variants in *PKD1* (78%) or *PKD2* (15%) encoding polycystin-1 and polycystin-2, respectively.^1^

Beyond renal manifestations, ADPKD is associated with early-onset hypertension and increased cardiovascular morbidity.^2^ Current management relies on lifestyle interventions and strict blood pressure (BP) control.^3^ Tolvaptan, the only approved disease-modifying therapy, slows cyst growth and chronic kidney disease progression in selected high-risk patients but is limited by cost and tolerability.

Polycystin-1 and polycystin-2 form a mechanosensitive complex on primary cilia. Defective polycystin signaling disrupts intracellular calcium homeostasis, promotes cystogenesis, and contributes to endothelial dysfunction.^4^ Endothelial cells lacking polycystin-1 or polycystin-2 display impaired shear stress–induced Ca^2+^-dependent nitric oxide release, leading to reduced endothelium-dependent vasodilation in patients with ADPKD.^5^ Recent findings suggest that stimulation of the type 5 dopaminergic receptor (DR5), expressed on endothelial and tubular epithelial primary cilia, may restore ciliary mechanosensitivity. In polycystin-deficient cells, exposure to dopamine, SKF-38393, or fenoldopam rescues intracellular Ca^2+^ signaling and reactivates nitric oxide synthesis in response to shear stress. Proposed mechanisms include restoration of cilia length and activation of ciliary Cav1.2 L-type Ca^2+^ channels, potentially compensating for defective polycystin-2 signaling^6^^,^^7^ (Supplementary Figure S1).

Early clinical studies with levodopa and dopamine demonstrated improvements in renal hemodynamics and endothelial function, although development was limited by the lack of selective vascular DR agonists.^8^^,^^9^^,^S1

Rotigotine, a transdermal dopaminergic agonist with DR5 activity, provides stable systemic exposure, requires no dose adjustment in chronic kidney disease, and has a favorable safety profile.S2

In the IMPROVE-PKD proof-of-concept study, transdermal rotigotine (4 mg/24 h for 2 months) improved endothelial function, flow-mediated nitric oxide release, and selected hemodynamic parameters in patients with ADPKD.S3 These vascular benefits, along with potential renal effects mediated by shared mechanisms in tubular epithelial cells, provide a strong rationale for long-term therapeutic evaluation.

The ETERNAL-PKD study was therefore designed to assess the safety of chronic rotigotine administration in patients with ADPKD, and explore its potential renal and cardiovascular effects to inform the design of a future phase 3 efficacy trial. We further hypothesized that DR5 stimulation could reduce BP and slow disease progression.

## Trial Design

### Study Population and Setting

This phase 2, open-label, randomized controlled trial was designed to evaluate the safety of rotigotine in adults aged 18 to 60 years with a confirmed diagnosis of ADPKD according to the Pei criteria. A total of 120 patients with ADPKD will be recruited from 4 French university hospitals (Amiens, Lille, Caen, and Rouen) over a 24-month enrollment period (NCT06291116). The study protocol was approved by a French ethical committee (CPP Sud Méditerranée IV, No. 2024-515734-32-00) on December 12, 2024. Patients must have received stable treatment with an angiotensin receptor blocker and/or tolvaptan for ≥4 weeks before inclusion. Patients must be normotensive or have adequately controlled hypertension (Table 1).

**Table 1:** Key inclusion and exclusion criteria

Inclusion criteria	Exclusion criteria
Adults aged 18 to 60 yrs with a confirmed diagnosis of ADPKD according to Pei criteria.	Stage 4 or 5 CKD (CKD-EPI estimated GFR < 30 ml/min per 1.73 m^2^)
Normotensive or well-controlled hypertensive patients defined as daytime ABPM < 135/85 mm Hg and/or 24-h mean ABPM < 130/80 mm Hg within the past 3 mo.	Kidney transplant recipients
Patients receiving a stable dose of ACE inhibitor or ARBs for ≥4 wk preceding enrollment, or patient documented to be unable to take ACE inhibitors or ARBs.	Patients undergoing dialysis
Patients receiving a stable dose of tolvaptan for ≥4 wk before enrollment.	History of myocardial infarction or stroke in the past 6 mo
Regulatory criteria:	Severe hepatic insufficiency (Child-Pugh class C)
Affiliation with or entitlement to a social security scheme.	Patients currently receiving or who have received within the 6 mo before the study, treatment with a dopaminergic agonist or antagonist (e.g., L-Dopa, neuroleptics, metoclopramide)
Written informed consent: patients who have read and understood the information letter and signed the informed consent form.	History of heart failure requiring hospitalization within the past 6 mo or known heart failure with a left ventricular ejection <30%
Effective method of contraception method for women of childbearing potential since ≥1 mo and during the study with a negative urine pregnancy test at inclusion.	Excessive alcohol consumption (>20 g/d)
Surgically sterile women	History of addictive behaviors, including gambling, compulsive shopping, or hypersexuality
Postmenopausal women	Drug addiction or suspected illicit drug use

ABPM, ambulatory blood pressure monitoring; ACE, angiotensin-converting enzyme; ADPKD, autosomal dominant polycystic kidney disease; ARB, angiotensin II receptor blocker; CKD-EPI, Chronic Kidney Disease-Epidemiology Collaboration equation; GFR, glomerular filtration rate.

### Study Procedures

**Figure 1:**
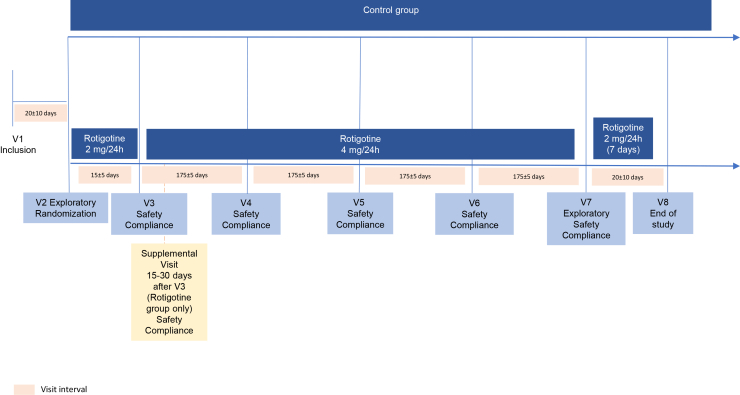
Study design. V, visit.

The study consists of 8 scheduled visits over 24 months, with 1 additional visit during the titration phase in the rotigotine group (Figure 1; Supplementary Table S1).

Clinical assessments, laboratory testing, BP measurements, imaging, quality-of-life evaluations, and treatment monitoring are performed according to a predefined schedule (Supplementary Methods).

### Objectives and End Points

The primary objective of this phase 2 trial is to evaluate the safety of rotigotine administered at 4 mg/24 h for 24 months. Safety will be assessed based on the occurrence of adverse reactions (ARs) and serious ARs during follow-up. The primary end point is the proportion of participants experiencing ≥1 serious AR during follow-up. Particular attention will be paid to known dopaminergic ARs, including severe application-site reactions, excessive somnolence, impulse control disorders, behavioral disturbances, fibrotic complications, and generalized skin reactions (Supplementary Table S2). These events may lead to treatment discontinuation (Supplementary Table S3). Adverse events will be graded by the investigator according to the Common Terminology Criteria for Adverse Events. Investigators will assess severity and causal relationship with study treatment.

Secondary objectives include evaluation of total kidney volume measured by magnetic resonance imaging using 3-dimensional segmentation, kidney function, urinary biomarkers of ADPKD progression, vascular parameters (e.g., BP), quality of life (ADPKD questionnaire), treatment compliance, and long-term treatment tolerability (Supplementary Table S2).

### Intervention

Patients will be randomized to one of the following 2 groups:

(i) Control group: standard of care management (cardiovascular risk factors, BP control, and tolvaptan when indicated) without rotigotine. Because the primary objective is safety evaluation, no placebo patch will be used.
(ii) Rotigotine group: standard-of-care management plus transdermal rotigotine (Neupro) according to the following schedule:

2 mg/24 h for 15 ± 5 days (visit V2–V3, titration phase).

4 mg/24 h from V3 to V7 (maintenance phase, 24 months).

2 mg/24 h for 7 days from V7 to V8 (tapering phase).

Statistical analysis, concomitant care and protocol modifications are presented in detail in the Supplementary Methods and Table S3.

## Discussion

The ETERNAL-PKD study is the first randomized controlled trial evaluating the long-term safety of a dopamine receptor agonist in ADPKD. The study is based on accumulating experimental and clinical evidence suggesting that DR5 activation may restore endothelial mechanotransduction impaired by polycystin deficiency.

In the IMPROVE-PKD study, rotigotine 4 mg/24 h significantly increased nitric oxide release and improved endothelial function. Although systemic hemodynamic parameters remained largely unchanged, improvements were observed in arterial wave reflection indices, including reductions in augmentation index and pulse pressure.S3 Similarly, chronic administration of the peripheral DR5 agonist, fenoldopam, improved vascular function and reduced BP in endothelial-specific *Pkd1* knockout mice.S3

Because DR5 is expressed in both endothelial and tubular epithelial cells, ETERNAL-PKD will provide important insights into the potential renal effects of chronic dopaminergic stimulation. These exploratory efficacy data will be essential for planning a future phase 3 trial. Rotigotine will be administered at low marketed doses (2–4 mg/d), and its AR profile is well characterized. Safety oversight will be reinforced by an independent Data Safety Monitoring Board, in accordance with regulatory requirements for phase 2 clinical trials.

Currently, tolvaptan remains the only approved therapeutic option for ADPKD. However, its efficacy in slowing kidney disease progression is modest, and its use is limited by tolerability issues. No existing strategy directly addresses the cardiovascular abnormalities frequently associated with ADPKD.

This is the first randomized trial to investigate the safety and potential efficacy of a DR agonist, rotigotine, in ADPKD, with a 24-month treatment period and a broad assessment of clinical, imaging, biological, and patient-reported outcomes. Importantly, we maintained inclusion criteria consistent with the IMPROVE-PKD trial,S3 which demonstrated a vascular effect of rotigotine 4 mg/24 h. The multicenter design enhances external validity and may facilitate the translation of the findings into clinical practice. Nonetheless, several limitations should be acknowledged. The open-label design may introduce bias, particularly for subjective end points. Magnetic resonance imaging will only be performed at baseline and 24 months, limiting the precision of total kidney volume progression assessment. The relatively small sample size and 2-year duration are appropriate for a phase 2 safety study but may be insufficient to detect robust effects on long-term renal outcomes. Finally, adherence challenges and dopaminergic ARs could impact treatment persistence and outcome interpretation.

Overall, this study aims to determine whether chronic dopaminergic stimulation may represent a relevant pharmacological target in ADPKD. Positive findings would support progression to a larger phase 3 efficacy trial and encourage the development of more selective peripheral DR5 agonists with improved tolerability.

## Disclosure

DG and JB are listed as inventors on a patent application based on the results described in IMPROVE-PKD study (application number WO2023EP74334). All the other authors declared no competing interests.

### Funding

The clinical trial was financed by the public grant Groupement Interregional pour la recherche Clinique et l’Innovation Nord-Ouest (grant number: PHRC-I 2022, API 22-14). The findings and conclusions in this report are those of the authors and do not necessarily represent the official position of the funding institution.

## Supplementary Material

Supplementary File (PDF) **Supplementary Method**s. Supplementary References. Figure S1. Simplified schematic representation of the effect of dopaminergic agonists in ADPKD. **Table S1.** Schematic diagram of patient participant timeline. **Table S2.** Primary, secondary objectives and end points. **Table S3.** Criteria for premature discontinuation of trial treatment and for premature study withdrawal.
