# Supplementary material for: Randomized Controlled Trial Evaluating Rotigotine Safety in PKD
Source: Kidney Int Rep. 2026 Jun 24;11(9):106682. doi: 10.1016/j.ekir.2026.106682 (PMC13427538; doi:10.1016/j.ekir.2026.106682)
Supplement: Supplementary File (PDF) — Supplementary Methods. Supplementary References. Figure S1. Simplified schematic representation of the effect of dopaminergic agonists in ADPKD. Table S1. Schematic diagram of patient participant timeline. Table S2. Primary, secondary objectives and end points. Table S3. Criteria for premature discontinuation of trial treatment and for premature study withdrawal. [file mmc1.pdf]

Supplementary materials

Randomized controlled trial evaluating rotigotine safety in polycystic kidney disease

Audrey Dumont[1], Adrien Cour[2], Muriel Quillard-Murraine[3], Margaux Van Wynsberghe[1], Sophie Ruault[4], Sabrina Prod'Homme[4], Estelle Houivet[5], Gabriel Choukroun[6], Cédric Renard[7], Clémence Béchade[8], Amandine Claudinot[9], François Glowacki[10], Philippe Puech[11], Nell Marty[4], Virginie Buchbach[4], Nathalie Donnadieu[12], Elise Duhamel[12], Jean-Nicolas Dacher[13], Dominique Guerrot[14], Jérémy Bellien[1].

Supplementary methods

Trial design

Recruitment and consent

Potentially eligible participants will be identified during routine follow-up visits or through telephone pre-screening conducted by the investigator nephrologist. Initial verbal consent may be obtained by phone followed by written informed consent prior to any study-specific procedures. During the inclusion visit, participants will receive comprehensive verbal and written information regarding the study's objectives, procedures, risks, constraints, and their rights. Adequate time will be provided for consideration before written consent is obtained. Separate consent will also be sought for plasma and urine biobanking as well as for DNA collection.

Study visits and procedures

V1-Inclusion visit: Conducted during a routine consultation. Eligibility criteria will be verified by the principal or sub-investigator. After informed consent is obtained, baseline data and additional biobanking consents will be collected.

V2-First exploratory and randomization visit (20±10 days after V1): Procedures include renal Magnetic Resonance Imaging (MRI) with non-contrast TKV (Total Kidney Volume) measurement, complete clinical examination, ADPKD-IS (Autosomal Dominant Polycystic Disease Impact Scale) quality of life questionnaire, blood and urine sampling and collection and urine pregnancy testing in non-menopausal women. Patients will then be randomized via Ennov software to the rotigotine or control group. Those allocated to rotigotine will receive

treatment at a starting dose of 2 mg/24 h via transdermal patch. Detailed instructions on patch application, storage, and accountability will be provided. Participants treated with rotigotine, will also begin maintaining a diary to document adherence and safety.

V3- V6 Follow-up visits: Scheduled 15±5 days after V2, between 15-30 days after V3 (rotigotine group only), then every 175±5 days. Each visit will include safety and adherence assessment (dispensed medication, diary review from participants treated with rotigotine), Adverse Events (AEs) and Serious Adverse Events (SAEs) reporting, concomitant medication review, clinical examination and blood and urine sampling. Urine pregnancy testing will be repeated in non-menopausal women. At V5, additional urine and plasma samples will be collected, a 24-hour ambulatory blood pressure monitoring (ABPM) performed and the ADPKD-IS questionnaire completed (also repeated at all visits except V3). Given the potential occurrence of visual disturbances under rotigotine treatment, a targeted questionnaire will be administered in the event of ocular symptoms, with referral for ophthalmological evaluation when clinically indicated. From V3 onwards, patients in the rotigotine arm will receive the maintenance dose of 4 mg/24 h, dispensed by the hospital pharmacy.

V7-Second exploratory visit (175±5 days after V6): Conducted in fasting conditions. Mirroring those of V5, including repeat renal MRI (same protocol as V2), and 24-h ABPM. Safety and adherence will be reassessed. If no safety concern is identified, rotigotine patients will receive the final treatment supply (7 patches, 2 mg/24h) for gradual dose tapering.

V8-End-of-study visit (20 ± 10 days after Visit V7): For rotigotine-treated patients, tolerance to treatment discontinuation will be assessed, including evaluation for depressive symptoms or apathy potentially related to dopamine agonist withdrawal. The patient diary will be collected. Long-term tolerability will be explored by asking: *"Would you tolerate receiving this treatment for the rest of your life?"* (Yes/No). If "*No*", the reason will be documented. Final AE/SAE collection will be performed for all participants.

Study procedures

Magnetic Resonance imaging

A non-contrast renal MRI will be performed to assess TKV using a 3D segmentation model. Multiple imaging sequences will be acquired including localizer sequences with and without breath-holding, T2-weighted TSE sequences in three planes, axial diffusion-weighted

sequences, coronal T1-weighted VIBE Dixon sequences and axial T1-weighted Dixon sequences. Renal MRI will be performed in the Radiology Departments of all participating centers, with blinded central analysis conducted by two study physicians. The total MRI acquisition time will not exceed 30 minutes. For patients receiving rotigotine 4 mg/24 h, the patch will be removed prior to MRI at V2 and V7 (as the Neupro® backing layer contains aluminium) and a new patch will be applied after image acquisition. A urine pregnancy test will be performed on non-menopausal participants just before the MRI.

Quality of life assessment

ADPKD-IS will be used to evaluate disease-specific quality of life scale. This validated instrument, developed based on patient input, consists of 18 items grouped into three conceptual domains: physical, emotional and fatigue.

Ambulatory blood pressure monitoring:

ABPM is a validated tool for the management of hypertension and is routinely performed annually for blood pressure monitoring in patients with ADPKD. Results from a 24-hour ABPM performed within the 3 months preceding V1, V5 and V7 will be collected.

Laboratory Assessments:

Biological samples will be collected by trained nursing staff at each site. Blood and urine tests will be performed at V2, V5, and V7, including complete blood count (CBC), platelet count, lipid profile, serum and urine electrolytes, urine albumin-to-creatinine ratio, total protein, blood glucose, urea, creatinine, and liver function tests. Urea, creatinine, aspartate aminotransferase (AST), and alanine aminotransferase (ALT) will be measured at V3, V4, and V6. Renal function will be estimated at visit V2 to V7, using eGFR (estimated glomerular filtration rate), calculated by the CKD-EPI formula, based on plasma creatinine (4 mL blood, heparinized tube).

Urinary biomarkers

Aliquots of urine collected at V2, V5 and V7 will be stored at -80°C in two cryotubes. For patients enrolled at external sites, samples will be shipped annually to the CIC-CRB biobank at University Hospital of Rouen. Biomarker concentrations will be determined by ELISA at INSERM U1096 Envi laboratory: copeptin (MBS2603322, MyBioSource), cAMP (581001, Cayman), MCP-1 (DCP00, R&D Systems) and AQP-2 (MBS2019939, MyBioSource).

Copeptin, cAMP and AQP-2 serve as biomarkers of the vasopressin pathway while MCP-1 is a pro-inflammatory and pro-fibrotic chemokine and an early indicator of disease severity in ADPKD.

Genetic Analysis

With participant consent, an additional 4 mL EDTA blood sample will be collected at Visit 2 (V2) for genetic analyses. Blood samples collected at external centers will be temporarily stored at –20°C and shipped annually to the CIC-CRB Inserm 1404 Biobank (Rouen University Hospital). Genomic DNA will be extracted using the QIAamp DNA Mini Kit (Qiagen). DNA concentration and purity will be determined using a NanoDrop 2000 spectrophotometer (Thermo Scientific). Extracted DNA will be aliquoted into two cryotubes and stored at -80°C at the CIC-CRB Inserm 1404 Biobank. The DNA biobank will be used for genotyping of *PKD1* and *PKD2* genes (performed by the reference laboratory in Brest) and for the analysis of DR5 receptor gene polymorphisms, potentially associated with rotigotine response.

Biomarker Analysis

With participant consent, additional 4 mL EDTA blood samples will be collected at Visits V2, V5, and V7 for the evaluation of renal and vascular function biomarkers. Following collection, samples will be centrifuged for 10 minutes at 1700 g and +4°C. Plasma will be aliquoted into two cryotubes to establish the plasma biobank. Samples collected at external centers will be stored at –80°C and shipped annually to the CIC-CRB Inserm 1404 Biobank (Rouen University Hospital). Biomarker analyses will be performed at the Inserm U1096 Laboratory.

Biological Sample Banking

Residual urine, plasma and DNA samples will be stored at -80°C for 5 years (urine, plasma biobank) or 20 years (DNA biobank).

Adverse Event Assessment and Causality Evaluation

All AEs will be assessed by the investigator for seriousness, intensity, outcome, and causal relationship with the investigational medicinal product, concomitant treatments, including tolvaptan, and study procedures. An AE will be considered related to rotigotine when a reasonable causal relationship with the investigational medicinal product cannot be excluded. Causality assessment will be based on the temporal relationship with rotigotine initiation, dose

escalation, interruption or discontinuation, the known safety profile of rotigotine, alternative explanations related to ADPKD or concomitant medications, and the clinical course of the event. The sponsor's pharmacovigilance unit will independently review causality and expectedness using the reference safety information for rotigotine transdermal patches. In case of disagreement between the investigator and the sponsor, both assessments will be retained in the safety documentation. To limit misclassification, typical ADPKD-related events, including kidney function decline, kidney cyst infection, intracystic hemorrhage, and nephrolithiasis, will be considered related to the underlying disease unless specific clinical features suggest otherwise

Statistical analysis

Sample size

This phase II trial is primarily designed for safety assessment, not efficacy. Secondary endpoints will generate preliminary efficacy data to inform a future phase III study, similar to prior ADPKD drug development programs[S4].

We aim to enrol 120 participants (60 per group). Assuming a 20% withdrawal rate, 96 evaluable patients (48 per group) are expected. With this sample size, incidence of Serious Adverse Reactions (SARs) can be estimated with a 95% CI of ±3.4% (assuming a 1.5% SAR rate in the rotigotine group). Similarly, we will be able to estimate the proportion of treatment discontinuation with a 95% CI of ±12.5%, assuming a 20% discontinuation rate, as observed at these doses in studies of restless legs syndrome[S2, S5].

Randomized and allocation

Randomization (1:1, stratified by center) will be generated by the Biostatistics Unit at Rouen University Hospital using SAS software and managed in Ennov. Investigators will receive allocations via secure email. The study is open-label; participants and clinicians will not be blinded. To reduce bias, renal MRI analyses will be centrally performed by two blinded physicians. Biological samples and clinical data will be pseudonymized.

Patient characteristics will be summarized overall and by randomization group (rotigotine *vs* control) using standard descriptive statistics: mean, SD, median, interquartile range, and range

for quantitative variables; and frequencies for qualitative variables. Comparisons will follow the intention-to-treat principle.

Primary outcome

The primary objective is to evaluate the long-term safety of rotigotine, defined as the occurrence of ≥1 SAR within 24 months. For each group, the proportion of patients experiencing at least one SAR will be estimated with corresponding 95% confidence intervals. Treatment discontinuation will also be evaluated, both overall and by cause (missed >2 doses/month, temporary interruption related to Adverse Reactions (ARs), permanent discontinuation due to SAR or other reasons). Long-term tolerability will be assessed by the proportion of patients responding "yes" versus "no" to the question: *Would you tolerate this treatment for the rest of your life?*

The mean number of ARs per patient will be compared between groups using either Student's t-test or Mann–Whitney *U* test as appropriate. Discontinuation rates will be compared using Chi-squared or Fisher's exact test.

Secondary outcomes

At 24 months, changes in eGFR, blood pressure (BP), TKV, ADPKD-IS score, and urinary biomarkers will be compared between groups using Student's t-test or Mann–Whitney U test, according to the distribution of data. Longitudinal trajectories from V2 to V7 will be analyzed using linear mixed-effects models with time and time-by-treatment interaction terms specified as fixed effects.

Subgroup analysis:

If sample size permits, exploratory subgroup analyses may be conducted according to progression status (rapid versus slow progressors), defined using the PROPKD score and baseline Mayo Clinic classification, in order to evaluate the effect of rotigotine on renal function and TKV.

Multivariable analysis:

Multivariable analysis will be performed to assess the association between rotigotine treatment and changes in renal function and TKV. Potential confounding factors will be taken into

account, including age, type of pathogenic variant (truncating and non-truncating variants in *PKD1* and *PKD2*), history of hypertension, concomitant tolvaptan treatment, and baseline renal function.

Concomitant care

Prohibited and permitted treatment/devices/procedures

Prohibited: participation in any other clinical trials for the duration of the study and 1 month after the end of the study, use of dopaminergic agonists/antagonists, or concomitant sedatives/ central nervous system depressants (such as benzodiazepines, antipsychotics, antidepressants, or antiemetic neuroleptics). Alcohol consumption must be limited ≤ 2 drinks per day. Rotigotine patches must be removed before cardioversion.

Permitted: all other treatments not listed above. No clinically significant drug interactions have been reported with rotigotine.

Modifications

Study Suspension and Early Discontinuation:

The sponsor, competent authorities, ethics committee, or the DSMB (Data Safety Monitoring Board) may suspend or terminate the trial for:

1. unexpected frequency or severity of ARs,

2. new safety concerns,

3. insufficient recruitment, or

4. inadequate data quality.

Study treatment premature discontinuation and premature study withdrawal:

Premature discontinuation of rotigotine may occur due to significant toxicity (e.g., sudden sleep episodes, persistent drowsiness, impulse control disorders or fibrotic complications, generalized skin reactions), deterioration of general health, major protocol violations, or pregnancy. A withdrawal visit will be performed as soon as possible. For discontinuation between V3 and V7, patients in the rotigotine arm will undergo a 7-day tapering course (2 mg/24 h). The investigator will notify the participant's general practitioner.

Participants who discontinue treatment after ≥6 months may remain in study follow-up ; otherwise, they will return to routine care. All patients will be monitored until AEs resolve or stabilize.

Data management and confidentiality

A secure, sponsor-supervised electronic data management system will be used in accordance with applicable regulations (GDPR EU 2016/679 and French data protection law No.78-17, CNIL MR-001). Study data will be recorded into electronic case report forms (eCRFs), with documented justifications for any missing values. All data will be pseudonymized and stored on a secure, encrypted server located in France. Access will be strictly limited to authorized personnel (investigators, sponsor representatives, and regulatory authorities) on a need-to-know basis.

Trial status

The trial protocol is version 1.3 (16th November 2024). A protocol amendment is currently in preparation in response to the reviewers' recent comments and requested modifications. Recruitment is expected to be completed by April 1st, 2029. This trial was recorded on clinicaltrials.gov (NCT06291116). The first participant was enrolled on May 12, 2026.

Data and Safety Monitoring Board

A DSMB, independent from the study and sponsor, comprising a nephrologist/physiologist, a pharmacologist, and a methodologist, oversees trial safety. The DSMB reviews safety data, protocol updates, and the investigator's brochure, issuing recommendations on continuation, modification, or termination of the study. Meetings are scheduled before enrollment, six months after first inclusion, annually, at study completion, and ad hoc on request of the sponsor in case of SAEs, particularly difficult to analyze or suspected unexpected serious adverse reaction (SUSAR) or if data is prone to modify the benefit/risk ratio.

Protocol amendments and ethical approval

The trial received ethical approval on 12 December 2024, ANSM (French National Agency for Medicines And Health Product Safety) authorization on 7 January 2025, and final CTIS approval on 13 January 2025. Any substantial protocol modifications (e.g., changes in study design, eligibility, or safety measures) require prior approval from the ethics committee, and ANSM.

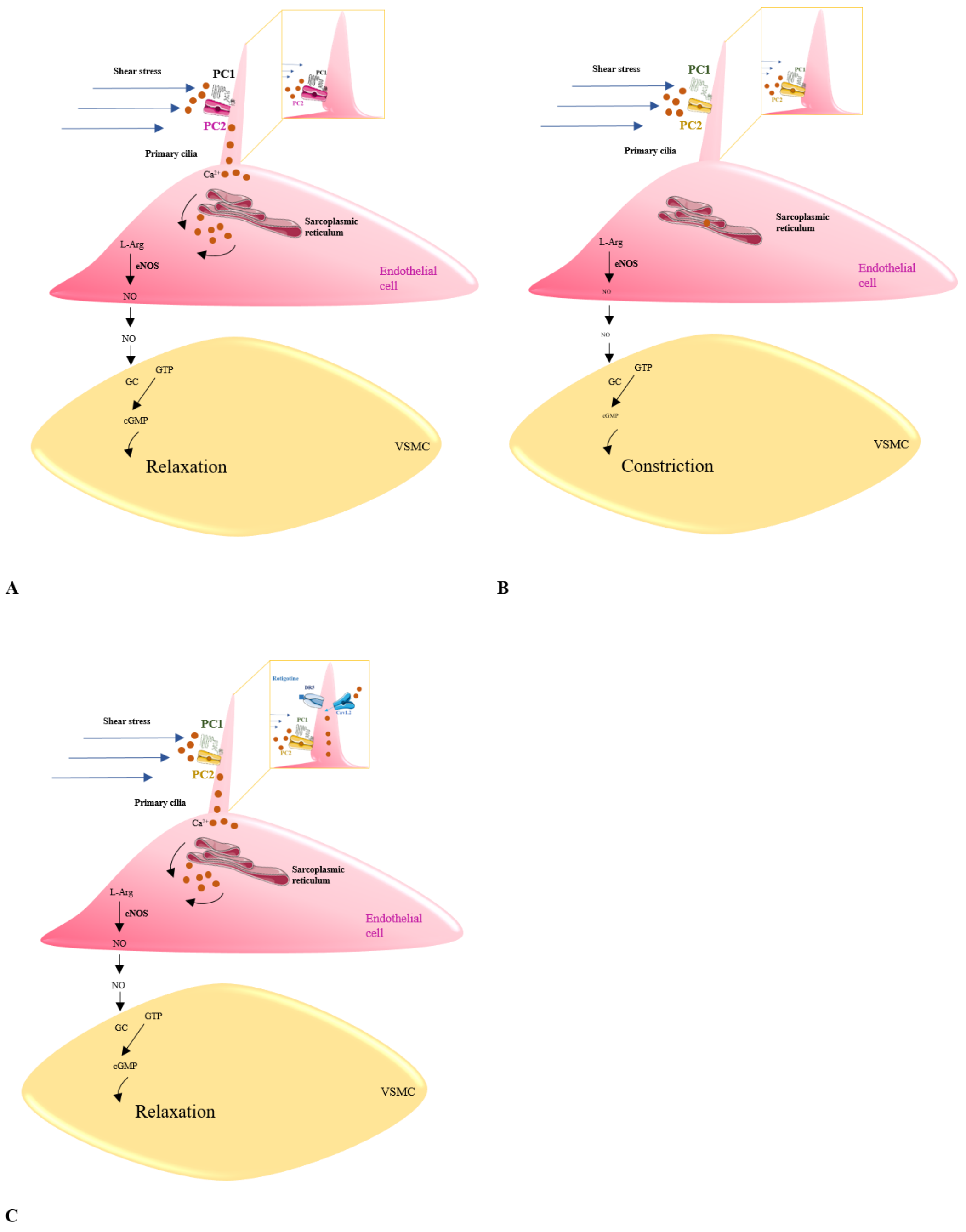

Figure S1: Simplified schematic representation of the effect of dopaminergic agonists in ADPKD.

A) Physiological conditions: under physiological conditions, functional PC1 and PC2 within endothelial cilia are associated with increased Ca^{2+} signaling in response to shear stress, resulting in NO release, cGMP production in VSMC and subsequent vasorelaxation.

B) ADPKD conditions: In ADPKD, dysfunctional PC1 and PC2 within endothelial cilia are associated with impaired Ca^{2+} signaling in response to shear stress, resulting in reduced NO release, and promoting vasoconstriction.

C) DR5 activation by rotigotine: Activation of the dopamine D5 receptor (DR5), sensitive to chemical and mechanical stimuli, restores ciliary Ca^{2+} influx in response to shear stress by promoting the opening of L-type voltage-dependent Ca^{2+} channels (Cav1.2). This leads to NO production and VSMC relaxation.

ADPKD: autosomal dominant polycystic kidney disease; DR: dopamine receptors; GC: guanylate cyclase; cGMP: cyclic guanosine monophosphate; NO: nitric oxide; PC1: polycystin 1; PC2: polycystin 2; VSMC: vascular smooth muscle cells.

Table S1. Schematic diagram of patient participant timeline

	Pre-screening	Visit V1 Inclusion	Visit V2 Exploratory et randomization (V1+20±10 days)	Visit V3[4] Follow-up (V2+15±5 days)	Visit V4 Follow-up M6 (V3+175±5 days)	Visit V5 Follow-up M12 (V4+175±5 days)	Visit V6 Follow-up M18 (V5+175±5 days)	Visit V7 Exploratory M24 (V6+175±5 days)	Visit V8 End of study visit (V7+20±10 days)
Patient information	✓								
Inform consent signing	✓	✓							
Clinical examination[1]		✓[2]	✓	✓	✓	✓[2]	✓	✓[2]	✓
Laboratory tests (including CBC/platelets, lipid profile, total protein, fasting glucose, urea, creatinine, electrolytes, liver function tests, urine electrolytes and urine albumin-to-creatinine ratio)			✓	✓ (Only: urea, creatinine, eGFR, AST, ALT)	✓ (Only urea, creatinine, eGFR, AST, ALT)	✓[2]	✓ (Only: urea, creatinine, eGFR, AST, ALT)	✓[2]	
Pregnancy test		✓	✓	✓	✓	✓	✓	✓	
Non-contrast renal MRI			✓					✓	
Treatment by rotigotine in the rotigotine group			✓	✓	✓	✓	✓	✓	
Ophtalmologic interview				✓[3]	✓[3]	✓[3]	✓[3]	✓[3]	✓[3]
Patient diary			✓	✓	✓	✓	✓	✓	
Collection for AEs/SAEs			✓	✓	✓	✓	✓	✓	✓
Assessment of adherence				✓	✓	✓	✓	✓	
Recording of concomitant			✓	✓	✓	✓	✓	✓	✓
ADPKD-IS questionnaire			✓		✓	✓	✓	✓	
24-h ABPM[2]		✓[2]				✓		✓	

Table S1 (continued). Schematic diagram of patient participant timeline

	Pre-screening	Visit V1 Inclusion	Visit V2 Exploratory et randomization (V1+20±10 days)	Visit V3[4] Follow-up (V2+15±5 days)	Visit V4 Follow-up M6 (V3+175±5 days)	Visit V5 Follow-up M12 (V4+175±5 days)	Visit V6 Follow-up M18 (V5+175±5 days)	Visit V7 Exploratory M24 (V6+175±5 days)	Visit V8 End of study visit (V7+20±10 days)
Urinary biomarkers of ADPKD progression (Copeptin, cAMP, MCP-1 and AQP-2)			✓			✓		✓	
Plasma/ Urine biobank			✓			✓		✓	
DNA biobank			✓						
Tolerance assessment									✓

[1]*Clinical examination at the inclusion visit includes collection of medical history, recording of usual treatments, measurement of weight and height, calculation of BMI (Body Mass Index), measurement of blood pressure and heart rate, and at a minimum, assessment of general condition, cardiovascular examination, and targeted examination based on any present symptoms.*

The clinical examination during follow-up visits includes recording of concomitant treatments, measurement of weight, calculation of BMI, measurement of blood pressure and heart rate, and at a minimum, assessment of general condition, cardiovascular examination, and targeted examination according to any present symptoms.

[2]*Assessment performed as part of routine clinical care. If the ABPM is older than three months at the time of inclusion, it must be repeated prior to inclusion.*

[3]*For participants in the rotigotine group, a clinical interview will be conducted to identify any symptoms suggestive of ophthalmologic involvement. If an abnormality is detected, an ophthalmology consultation will be scheduled.*

[4]*A follow-up visit between Day 15 and Day 30 after V3 will be conducted for patients in the rotigotine group to assess the patient's general condition and to ensure that no adverse events have occurred that would preclude continuation of treatment. Treatment compliance will also be evaluated.*

ABPM: ambulatory blood pressure monitoring; AEs: adverse events; ALT : alanin aminotransferase ; AQP-2: aquaporin 2; AST : aspartate aminotransferase ; cAMP: cyclic adenosine monophosphate; GFR: estimated glomerular filtration rate; MCP-1: monocyte chemoattractant protein-1; SAEs : serious adverse events.

Table S2: Primary, secondary objectives and end points.

**Objective**	**End point**
**Primary objective**	**Primary endpoint**
The primary objective of the present study is to evaluate the safety of rotigotine administered at a dose of 4 mg/24h over a 24-month period in patients with ADPKD	Safety is defined by the occurrence of ARs and SARs over a 24-month period. The primary safety endpoint is based on the proportion of participants who experience at least one SAR during the 24-month follow-up period of the study, such as the occurrence of serious application site reactions or certain behavioral disorders
**Secondary objectives**	**Secondary endpoints**
To demonstrate that chronic administration of rotigotine (4 mg/24 hours) for 24 months slows the progression of ADPKD as assessed by changes in total kidney volume (TKV) and renal function compared to patients not receiving rotigotine	a. To assess the change over 24 months in TKV measured by MRI and adjusted for height.<br><br>b. To assess the change over 24 months in eGFR using the CKD-EPI formula
To determine whether rotigotine reduces blood pressure compared to patients not receiving the treatment	To assess the change over 24 months in blood pressure using 24-hour ABPM
To assess whether rotigotine improves quality of life relative to untreated patients	To assess the change over 24 months in quality of life using the ADPKD-IS questionnaire
To evaluate the effect of rotigotine on urinary biomarkers associated with ADPKD progression	To assess the change over 24 months in urinary biomarkers of ADPKD progression (copeptin, cAMP, MCP-1, and AQP-2)
To assess treatment adherence over the 24-month period	To assess treatment adherence over 24 months through the calculation of the discontinuation rate
To evaluate the long-term tolerability of rotigotine over 24 months of treatment.	To assess the proportion of patients who answered "yes" (and respectively "no") to the question: "Would you tolerate this treatment for the rest of your life?

ADPKD: autosomal dominant polycystic kidney disease; ADPKD-IS: ADPKD-Impact scale; ABPM : ambulatory blood pressure monitoring ; ALT : alanine aminotransferase ; AQP-2: aquaporin-2; ARs: adverse reactions; AST : aspartate aminotransferase ; cAMP: cyclic adenosine monophosphate; eGFR:estimated glomerular filtration rate; MCP-1: monocyte chemoattractant protein-1, MRI: magnetic resonance imaging; *SARs: Serious adverse reactions; TKV: total kidney volume.*

Table S3. Criteria for premature discontinuation of trial treatment and for premature study withdrawal.

**Criteria for premature discontinuation of the investigational treatment**	Toxicity, based on medical advice and/or the participant's decision, such as:<br>• Sudden onset of sleep episodes or persistent drowsiness<br>• Persistent impulse control disorders or related disorders<br>• Fibrotic complications<br>• Generalized skin reactions<br><br>Other reasons
	Deterioration in the general condition of the participant
	Major protocol violation
	Pregnancy
	Breastfeeding
**Criteria for premature study withdrawal:**	Participant's decision (no justification required, data prior to withdrawal retained)
	Investigator's decision in participant's best interest (e.g., SAE, incompatible treatment)
	Death
	Lost to follow-up

Consort checklist

Section / Topic	No	CONSORT 2025 checklist item description	Reported on page no.
**Title and abstract**			
Title and structured abstract	1a	Identification as a randomised trial	Page 1
	1b	Structured summary of the trial design, methods, results, and conclusions	No abstract in Research letter
**Open science**			
Trial registration	2	Name of trial registry, identifying number (with URL) and date of registration	Page 4
Protocol and statistical analysis plan	3	Where the trial protocol and statistical analysis plan can be accessed	Page 4<br>Supplementary materials, 2-7
Data sharing	4	Where and how the individual de-identified participant data (including data dictionary), statistical code and any other materials can be accessed	Page 7
Funding and conflicts of interest	5a	Sources of funding and other support (e.g., supply of drugs), and role of funders in the design, conduct, analysis and reporting of the trial	Page 7
	5b	Financial and other conflicts of interest of the manuscript authors	Page 7
**Introduction**			
Background and rationale	6	Scientific background and rationale	Page 2-3
Objectives	7	Specific objectives related to benefits and harms	Page 4
**Methods**			
Patient and public involvement	8	Details of patient or public involvement in the design, conduct and reporting of the trial	Page 4
Trial design	9	Description of trial design including type of trial (e.g., parallel group, crossover), allocation ratio, and framework (e.g., superiority, equivalence, non-inferiority, exploratory)	Page 4
Changes to trial protocol	10	Important changes to the trial after it commenced including any outcomes or analyses that were not prespecified, with reason	Supplementary materials, 9
Trial setting	11	Settings (e.g., community, hospital) and locations (e.g., countries, sites) where the trial was conducted	Page 4
Eligibility criteria	12a	Eligibility criteria for participants	Page 4
	12b	If applicable, eligibility criteria for sites and for individuals delivering the interventions (e.g., surgeons, physiotherapists)	4<br>Supplementary materials, page 2
Intervention and comparator	13	Intervention and comparator with sufficient details to allow replication. If relevant, where additional materials describing the intervention and comparator (e.g., intervention manual) can be accessed	Page 5<br>Supplementary materials, page 6
Outcomes	14	Pre-specified primary and secondary outcomes, including the specific measurement variable (e.g., systolic blood pressure), analysis metric (e.g., change from baseline, final value, time to event), method of aggregation (e.g., median, proportion), and time point for each outcome	Page 4-5<br>Supplementary materials, page 7
Harms	15	How harms were defined and assessed (e.g., systematically, non-systematically)	Page 5

Sample size	16a	How sample size was determined, including all assumptions supporting the sample size calculation	Supplementary materials, page 6
	16b	Explanation of any interim analyses and stopping guidelines	Supplementary materials, page 8
Randomisation:			
Sequence generation	17a	Who generated the random allocation sequence and the method used	Supplementary materials, page 6
	17b	Type of randomisation and details of any restriction (e.g., stratification, blocking and block size)	Supplementary materials, page 6
Allocation concealment mechanism	18	Mechanism used to implement the random allocation sequence (e.g., central computer/telephone; sequentially numbered, opaque, sealed containers), describing any steps to conceal the sequence until interventions were assigned	Supplementary materials, page 6
Implementation	19	Whether the personnel who enrolled and those who assigned participants to the interventions had access to the random allocation sequence	Supplementary materials, page 6
Blinding	20a	Who was blinded after assignment to interventions (e.g., participants, care providers, outcome assessors, data analysts)	Page 4<br><br>Supplementary materials, page 6
	20b	If blinded, how blinding was achieved and description of the similarity of interventions	/
Statistical methods	21a	Statistical methods used to compare groups for primary and secondary outcomes, including harms	Supplementary materials, pages 6-7
	21b	Definition of who is included in each analysis (e.g., all randomised participants), and in which group	Supplementary materials, pages 6-7
	21c	How missing data were handled in the analysis	Supplementary materials, page 9
	21d	Methods for any additional analyses (e.g., subgroup and sensitivity analyses), distinguishing prespecified from post-hoc	Supplementary materials, pages 6-7
**Results**			
Participant flow, including flow diagram	22a	For each group, the numbers of participants who were randomly assigned, received intended intervention, and were analysed for the primary outcome	Supplementary materials, page 6
	22b	For each group, losses and exclusions after randomisation, together with reasons	Supplementary materials , page 6-8
Recruitment	23a	Dates defining the periods of recruitment and follow-up for outcomes of benefits and harms	Supplementary materials, page 2
	23b	If relevant, why the trial ended or was stopped	Supplementary materials, page 8
Intervention and comparator delivery	24a	Intervention and comparator as they were actually administered (e.g., where appropriate, who delivered the intervention/comparator, how participants adhered, whether they were delivered as intended [fidelity])	Page 4
	24b	Concomitant care received during the trial for each group	Page 4
Baseline data	25	A table showing baseline demographic and clinical characteristics for each group	Not applicable
Numbers analysed,<br><br>outcomes and estimation	26	For each primary and secondary outcome, by group:<br><br>• the number of participants included in the analysis	Supplementary materials, page 6-7

		• the number of participants with available data at the outcome time point<br>• result for each group, and the estimated effect size and its precision (such as 95% confidence interval)<br>• for binary outcomes, presentation of both absolute and relative effect size	
Harms	27	All harms or unintended events in each group	Supplementary materials, page 6-7
Ancillary analyses	28	Any other analyses performed, including subgroup and sensitivity analyses, distinguishing pre-specified from post-hoc	Supplementary materials, page 6-7
**Discussion**			
Interpretation	29	Interpretation consistent with results, balancing benefits and harms, and considering other relevant evidence	Page 6
Limitations	30	Trial limitations, addressing sources of potential bias, imprecision, generalisability, and, if relevant, multiplicity of analyses	Page 6

Supplementary references

S1. Kathem S, Mohieldin AM, Abdul-Majeed S, *et al.* Ciliotherapy: a novel intervention in polycystic kidney disease. *J Geriatr Cardiol.*2014;11:63-73. https://doi.org/10.3969/j.issn.1671-5411.2014.01.001.

S2. Bogan RK. From bench to bedside: An overview of rotigotine for the treatment of restless legs syndrome. *Clin Ther.*2014;36:436-455. https://doi.org/10.1016/j.clinthera.2014.01.021.

S3. Dumont A, Hamzaoui M, Groussard D, *et al*. Chronic endothelial dopamine receptor stimulation improves endothelial function and hemodynamics in autosomal dominant polycystic kidney disease. *Kidney int.* 2024; 106(6):1158-1169. https://doi.org/10.1016/j.kint.2024.08.020.

S4. Perrone RD, Abebe KZ, Watnick TJ, *et al.* Primary results of the randomized trial of metformin administration in polycystic kidney disease (TAME PKD). *Kidney Int.*2021;100: 684-696. https://doi.org/10.1016/j.kint.2021.06.013.

S5. Dohin E, Högl B, Ferini-Strambi L, Schollmayer E, *et al.* Safety and efficacy of rotigotine transdermal patch in patients with restless legs syndrome: a post-hoc analysis of patients taking 1 - 3 mg/24 h for up to 5 years. *Expert Opin. Pharmacother.* 2013;14:15-25. https://doi.org/10.1517/14656566.2013.758251.
